# The use of diagnostic ultrasound by primary care physicians in Switzerland – a cross-sectional study

**DOI:** 10.1186/s12875-024-02491-5

**Published:** 2024-07-06

**Authors:** Nico Zumstein, Christoph Merlo, Stefan Essig, Reto Auer, Kali Tal, Roman Hari

**Affiliations:** 1 Institute of Primary Health Care (BIHAM), University of Bern, Bern, Switzerland; 2 Dean’s Office, Medical Faculty, University of Bern, Bern, Switzerland; 3 Center for Primary and Community Care, University of Lucerne, Lucerne, Switzerland; 4 Center for Primary Care and Public Health (Unisanté), University of Lausanne, Lausanne, Switzerland; 5 Swiss Sentinel Surveillance Network, Federal Office of Public Health, Bern, Switzerland

**Keywords:** Ultrasound, Point-of-care, Primary care, General practitioners, Indications, Training, Switzerland

## Abstract

**Background:**

Diagnostic ultrasound has become a bedside tool widely available to many primary care physicians (PCPs) in Europe. It is often used as point-of-care ultrasonography (POCUS) in this setting. In Switzerland, certain POCUS examinations are listed as learning objectives in existing ultrasound training programs (we defined these examinations as swissPOCUS = sPOCUS). Ultrasound performed by PCPs can lead to faster diagnostic workup and reduce referral to secondary care units. However, adequate training is crucial to guarantee high quality. To guide the development of ultrasound training programs for PCPs, this study explores the use of ultrasound in primary care in Switzerland.

**Methods:**

This was a cross-sectional study. We invited PCPs from the Swiss practice-based research network “Sentinella” to collect data on the first 5 daily ultrasounds they ordered or performed themselves. Participating PCPs collected data for 3 months – divided into 4 groups to account for seasonal differences.

**Results:**

Out of 188 PCPs invited, 81.9% provided data through an initial questionnaire. 46.8% provided data on 1616 ultrasounds. 56.5% of PCPs had access to ultrasound machines, while 29.8% had completed formal training. 77% of the reported ultrasounds were self-performed; 27% of the reported scans (35% of all self-performed scans) were performed by PCPs with incomplete or no formal training.

The main areas of interest were the abdominal (57.9%) and the musculoskeletal (22%) region. 36.9% of reported examinations were sPOCUS exams. Among PCPs with access to US machines, the percentages of referred examinations were similar for sPOCUS (11.9%) and non-sPOCUS (11.3%) indications. However, some sPOCUS musculoskeletal ultrasounds were often referred (e.g. tendon/ligament/muscle injuries or cutaneous/subcutaneous tumour).

**Conclusion:**

Most Swiss PCPs had access to ultrasound equipment and performed a majority of both sPOCUS and non-sPOCUS scans themselves, often without or with incomplete training. This reflects the fact that POCUS was only recently introduced in Switzerland. There is a need for easily accessible POCUS training programs aimed at PCPs in Switzerland.

Training courses for PCPs should focus on abdominal and musculoskeletal ultrasound, because these were the most common sites for scans, and because some sPOCUS musculoskeletal examinations showed a particularly high percentage of referral.

**Supplementary Information:**

The online version contains supplementary material available at 10.1186/s12875-024-02491-5.

## Background

Diagnostic ultrasound has become a bedside tool widely available to many primary care physicians (PCPs) in Europe [1–6]. Ultrasound examinations in primary care lead to faster diagnostic workup and improve clinical decision-making [7]. They reduce procedure-related complications when used to guide diagnostic procedures [8]. Furthermore, ultrasound performed directly by PCPs at the point-of-care improves patient’s experience of care, their confidence in diagnosis and lead to a reduction of referral to secondary care units [7, 9, 10]. Evidence from secondary healthcare showed that use of ultrasound at the point-of-care can lead to a more rational use of healthcare resources [11, 12].

In recent years point-of-care ultrasonography was introduced as a new, more clinically driven way to use ultrasound. It moves away from comprehensive imaging techniques (such as comprehensive ultrasound, CT or MRI) and uses ultrasound for focused examinations at bedside to answer binary, clinically driven questions (e.g. gallbladder stones yes/no, obstructive uropathy yes/no) [2, 13, 14]. If we wish to refer to this definition of point-of-care ultrasonography we will use the term POCUS in the following text.

This focused approach is inherently very close to typical applications for ultrasound in primary care. According to a Danish study from 2020 73% of ultrasounds in primary care are used to confirm or disconfirm one specific tentative diagnosis [7]. Focused POCUS scans performed by PCPs were reported to have higher diagnostic accuracy than more comprehensive scans [4].

Both comprehensive and POCUS ultrasounds are highly user dependant and adequate training is crucial to guarantee high quality in diagnostic ultrasound. A systematic literature review from 2020 showed a vast variety of training programs for PCPs worldwide for both POCUS and comprehensive approaches to ultrasound [2]. A survey based study from 2016 shows significant differences in use, organisation and training within 12 European countries and states a lack of training as one of the most important barriers to the use of ultrasound in primary care [1]. Two Swiss studies showed that around half (49%) of PCPs in Switzerland use diagnostic ultrasound (both POCUS and comprehensive), but only 18% of PCPs also have a national certificate that ensures quality standards [15, 16]. This lack in accreditation and formal training is likely due to high requirements needed to obtain and to maintain the Swiss certificate on comprehensive abdominal ultrasound and also the shortage of accessible and primary care oriented POCUS training [15, 17].

Ultrasound training for PCPs should be directed to the most common clinical questions that can be answered at the point-of-care. In 2020 a survey was conducted on 61 PCPs attending ultrasound courses of the Swiss society of ultrasound in medicine (SGUM). Results show a wide variety of indications in the PCPs current ultrasound use. Indications in the abdominal region (69%) were the most frequent among them, followed by rectal/vaginal (15%, mainly in female patients) and musculoskeletal (8%) indications [15]. These results correspond with similar results from further studies in other countries, where abdominal scans were predominant in ultrasound scans performed by PCPs [18, 19]. In 2020 a consensus paper from Denmark stated mainly scanning modalities within the musculoskeletal (8/30), abdominal (5/30) and obstetric (5/30) area as learning goals for future POCUS training programs aimed at PCPs [20]. To this point there is no sufficient data which clinical questions Swiss PCPs (and PCPs worldwide) are trying to answer trough ultrasound.

With several studies supporting the advantages of ultrasound—especially focused POCUS examinations—in primary care but also the need for specific training, we must better understand the current state and requirements of ultrasound use in general practice. We therefore aimed to establish a solid empirical foundation for a high quality ultrasound training tailored for primary care. More specifically, we aimed to evaluate Swiss PCP’s access to ultrasound, their current state of ultrasound training and the indications of ultrasound scans performed or referred over a period of one year. Secondly, we aimed to explore current referral practice and the prevalence of POCUS use in PCPs who have access to ultrasound.

## Methods

### Study design

This cross-sectional study assessed Swiss PCP’s use of ultrasound within the practice-based research network “Sentinella”. PCPs filled out a baseline questionnaire and then entered data on ultrasounds they ordered or performed during a period of three months.

### Study population

This study was conducted within the Swiss Sentinella network and included all PCPs who belonged to Sentinella during the study period. The Sentinella network is a co-project of dedicated Swiss PCPs, the Federal Office of Public Health in Switzerland (BAG), and the university institutes for family medicine. Sentinella, introduced in 1986 to collect surveillance data on communicable diseases (especially influenza), now collects data to answer research questions about primary care. From 150 to 250 general internal medicine and paediatrics family practices participate in Sentinella and report anonymous patient data each week [21]*.* PCPs are compensated annually for collecting routine data, paid by the Federal Office of Public Health (BAG), but they are not required to participate in Sentinella surveys.

The PCPs were recruited for this study by the BAG through the Sentinella online tool. Communication took place through the Sentinella online tool and e-mail. E-mails were sent (anonymously for the authors) via a BAG distribution list.

### Context

In Switzerland, billing ultrasounds to the insurance requires a completed specialist training for any specialisation and an additional certificate by the Swiss Society of Ultrasound in Medicine. At the time of this study existed 14 different, specialised certificates in point-of-care ultrasonography (e.g. emergency sonography, sonography of the musculoskeletal system, different types of cardiac ultrasound etc.). None of these are aimed specifically at PCPs. Alternatively, there is a certificate in comprehensive ultrasound of the abdomen. Until 2018, when the above mentioned POCUS certificates were introduced this certificate in comprehensive ultrasound of the abdomen was the only way to obtain formal training and certification for abdominal ultrasound for Swiss PCPs. Therefore it is not unusual for Swiss PCPs to be performing comprehensive as well as focused ultrasound examinations. All of the above certificates require physicians to participate in formal training, which often lasts several days, perform a minimum number of ultrasound examinations under supervision (provided by a holder of the certificate, which is usually a specialist in internal medicine or radiology) and pass a summative exam [17, 22].

Since January 2022, it has been mandatory in Switzerland to hold the POCUS emergency sonography certificate to qualify as a specialist in general internal medicine (the specialisation needed to practice as PCP for adult patients).

### Inclusion criteria

All ultrasounds ordered or performed by PCPs were eligible except for obstetric and cardiac ultrasounds. These ultrasounds are almost exclusively performed by gynaecologists and cardiologists in Switzerland [15].

### Data collection

Before we began the study, we piloted and refined our questionnaires. We selected three PCPs, who worked with us to improve usability, fix logical errors and add any missing survey items. In 2021, when the study began, Sentinella physicians were asked to fill out a baseline questionnaire on Survey Monkey that asked them about their access to ultrasound machines, their experience and training in ultrasonography and how often they performed ultrasounds. They were then asked to assess their ability to diagnose or exclude certain pathologies via ultrasound, describe the areas in which they most needed more ultrasound training and indicate their level of interest in taking more training courses aimed at primary care physicians. The baseline questionnaire was developed for this study (see supplementary File 1 “baseline questionnaire”) and was based on literature that investigated similar topics [8, 15, 23, 24].

**Fig. 1:**
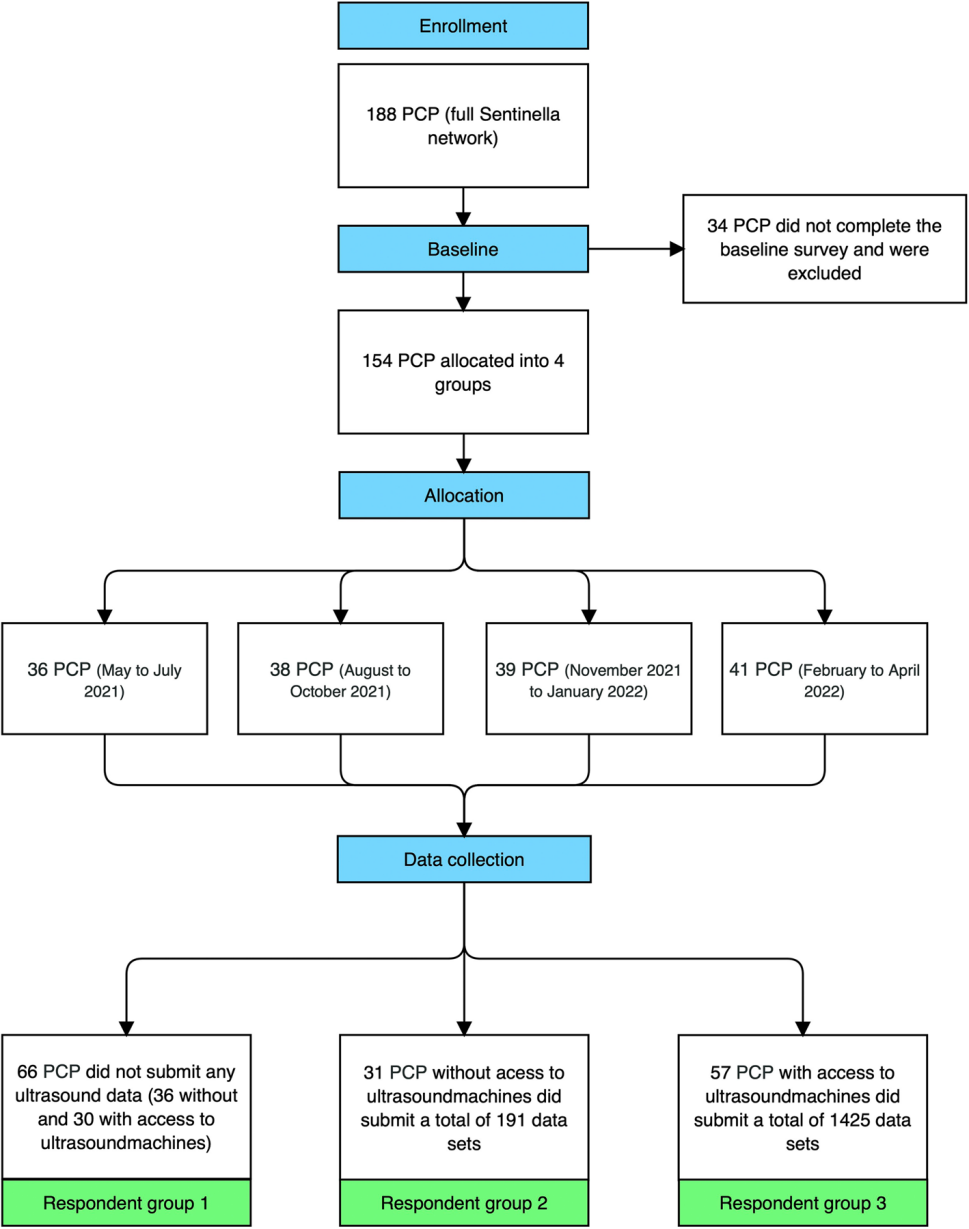
Overview of course of study

From June 1st 2021 until Mai 30th 2022, Sentinella physicians were asked to provide data on the first 5 ultrasounds they ordered or performed each day over a 3-month period. To control for seasonal differences in ultrasound indications, participating PCPs were divided into four equally distributed groups, each of which started collecting data at a different time of year (see Fig. 1). PCPs were stratified by their use of ultrasound machines and experience and training in ultrasonography.

PCPs were asked to provide the following data for each ultrasound exam: patient demographics (year of birth and gender); clinical question(s); region(s) of interest; urgency level; and whether the exam was performed in-house by the PCP or referred to a secondary care institution. For every ultrasound, PCPs were asked if the exam confirmed or excluded the clinical question, whether further inquiry was necessary, or if there were incidental findings. If the PCP performed the ultrasound, they were asked to report the length of the examination and type of billing. Again, the questionnaire was developed for this study (see supplementary File 2 “main questionnaire”) and followed recommendations from the literature on similar topics [8, 15, 23, 24].

PCPs entered data into an online survey housed on the Sentinella platform, which PCPs routinely use to enter other data.

### Data analysis

The primary outcome of interest was PCP’s access to ultrasound, their current state of ultrasound training and the indications of ultrasound scans performed or referred.

Secondary outcomes included differences in proportions of ultrasounds performed by PCPs or ordered externally, and relative prevalence of POCUS scans. Since the aim of this secondary outcome was to investigate the use of ultrasound within the existing Swiss ultrasound training environment we introduced the term sPOCUS (swissPOCUS) and defined it as a focused ultrasound examination with a clinical question listed as a learning objective in an existing Swiss POCUS training program at the time of this study [22]. Scans with clinical questions that were not listed as learning goal in an existing Swiss POCUS training program at the time of this study or more comprehensive scans (e.g. full abdominal scans) were defined as non-sPOCUS (non-swissPOCUS). For such non-sPOCUS examinations Swiss PCPs either have to obtain training in comprehensive ultrasound or there is no formal training program. If we refer to both (sPOCUS and non-sPOCUS) we use the broad term ultrasound.

The subgroup analysis for the secondary outcome focused on ultrasounds reported by PCPs with access to ultrasound and thus excluded data reported by PCPs who had no access to ultrasound machines. Also, because a specific POCUS training for paediatricians has been available since 2018, we wanted to specifically investigate the use of ultrasound by PCPs for adult patients. Therefore, we also excluded data sets reported by participating paediatricians in this analysis. To avoid confusion when we report results, we refer to PCPs who are not paediatricians as General practitioners (GPs). When we wish to indicate the group of GPs and paediatricians are meant, we use the broader term PCPs.

We present categorical data as raw case numbers or summarised as frequencies and proportions. To summarise continuous variables, we used means and standard deviations (SD). We performed descriptive analysis and calculations in Microsoft Excel (Version 16.5). Because our main results concern descriptive data and outcome data for referred ultrasound were largely missing due to the way data was collected, we didn’t perform any statistical analysis.

## Results

### Participants and responses

Figure 1 provides an overview of the study. The baseline questionary was sent to all PCPs in the Sentinella network during the data collection period (*n* = 188): 154 PCPs completed the baseline questionnaire (response rate: 81.9%) and were assigned to one of four groups for data collection; 34 PCPs did not fill out the baseline questionnaire and were excluded from further data collection.

After data collection we divided the 154 PCPs that were included for data collection into 3 groups (referred to as respondent group 1–3 in Fig. 1 and in the following text). Respondent group 1 contains all PCPs (*n* = 66) that have not reported any ultrasound data (30/66 = 45.5% with access to ultrasound machines). Respondent group 2 contains all PCPs (*n* = 31) which have collected data, but didn’t have access to ultrasound machines. Respondent group 3 contains all PCPs (*n* = 57) that have collected data and had access to ultrasound machines. Resulting to 64.8% (57/88) PCPs having access to ultrasound machines in respondent group 2 and 3. We will refer to this grouping in the further course of the text.

Respondent group 2 and 3 (*n* = 88; response rate: 46.8%) collected 1616 data sets on ultrasounds they performed or referred during the 3 months they collected data (average: 18.4 data sets per PCP).

Respondent group 2 (*n* = 31; 35.2% of PCPs that collected ultrasound data) submitted 11.8% of data sets (*n* = 191), averaging 6.2 data sets per PCP.

Respondent group 3 (*n* = 57; 64.8% of PCPs that collected ultrasound data) submitted 88.2% of all data sets (*n* = 1425), averaging 25 data sets per PCP.

### PCP’s access to ultrasound and their current state of ultrasound training

Table 1 summarizes the characteristics of PCPs who completed the baseline questionnaire (*n* = 154; respondent group 1, 2 and 3).

**Table 1:** Characteristics of PCPs that completed the baseline questionnaire

		Access to US-Machine		No access to US-Machine		Total	
PCPs mean age (in years)		54	*-*	55	*-*	54.4	*-*
Total		87	*56.5%*	67	*43.5%*	154	*100%*
Gender	Female	19	*12.3%*	24	*15.6%*	43	*27.9%*
	Male	65	*42.2%*	39	*25.3%*	104	*67.5%*
	Unknown	3	*1.9%*	4	*2.6%*	7	*4.5%*
Form of practice	Group practices	59	*38.3%*	30	*19.5%*	89	*57.8%*
	Single practices	25	*16.2%*	33	*21.4%*	58	*37.7%*
	Unknown	3	*1.9%*	4	*2.6%*	7	*4.5%*
Discipline	Pediatrician	9	*5.8%*	10	*6.5%*	19	*12.3%*
	GP with pediatric patients	43	*27.9%*	25	*16.2%*	68	*44.2%*
	GP without pediatric patients	31	*20.1%*	28	*18.2%*	59	*38.3%*
	Unknown	4	*2.6%*	4	*2.6%*	8	*5.2%*
US-Training	Pediatrician with completed training	11	*7.1%*	0	*0.0%*	11	*7.1%*
	Pediatrician with some training	0	*0.0%*	0	*0.0%*	0	*0.0%*
	Pediatrician without training	0	*0.0%*	8	*5.2%*	8	*5.2%*
	GP with completed training	35	*22.7%*	0	*0.0%*	35	*22.7%*
	GP with some training	15	*9.7%*	5	*3.2%*	20	*13.0%*
	GP without training	22	*14.3%*	46	*29.9%*	68	*44.2*
	Unknown	4	*2.6%*	8	*5.2%*	12	*7.8%*

Percentages refer to the total number of 154 completed baseline questionnaires

*US* Ultrasound

Of these, 56.5% of PCPs had access to ultrasound machines in or outside their practice.

38.3% were GPs who treated only adult patients, 44.2% were GPs who also treated paediatric patients (< 16 years of age), 12.3% of PCPs were paediatricians. Of PCPs with access to ultrasound machines, 69% performed up to 5 ultrasounds per week, 20.7% performed 5–10 ultrasounds per week, and 5.7% performed 10–15 ultrasounds per week. No participant performed > 15 ultrasounds per week (4.6% did not answer this question). 29.8% of all participating PCPs had completed formal ultrasound training, 13% had some formal training but did not complete it, and 49.4% reported no formal training.

Table 2 shows an overview of all reported ultrasounds, stratified by PCP’s state of ultrasound training and their access to ultrasound machines (data from respondent group 2 and 3 was used).

**Table 2:** Characteristics of reported ultrasounds

PCPs		Ultrasounds							
		Total		Self-performed		Referred		Unknown	
Total (*n* = 88)		1616	*100%*	1245	*77%*	343	*21.2%*	28	*1.7%*
Training	Completed (*n* = 46)	849	*52.2%*	809	*50.1%*	27	*1.6%*	13	*0.8%*
	None or uncompleted (*n* = 42)	767	*47.5%*	436	*27%*	316	*19.6%*	15	*0.9%*
Access to ultrasound machines	No (*n* = 31) = Respondent group 2	191	*11.8%*			189	*11.7%*	2	*0.1%*
	Yes (*n* = 57) = Respondent group 3	1425	*88.2%*	1245	*77%*	154	*9.5%*	26	*1.6%*

Stratified by two criteria: PCPs with or without access to ultrasound machines and with or without completed formal training (each group contains 100% of all reported ultrasounds)

PCPs reported a total of 1616 ultrasounds. 77% (*n* = 1245) of the reported ultrasounds were self-performed and 21.2% (*n* = 343) ultrasounds were referred.

Of the 1245 ultrasounds that were self-performed, 35% (*n* = 436) were performed by PCPs with incomplete or no formal training (equals 27% of all reported scans).

The PCPs who had completed formal training (*n* = 46) reported 52.5% (*n* = 849) of all ultrasounds. They self-performed 95.3% (*n* = 809) of these ultrasounds (or 50.1% of all reported ultrasound).

Of all 1616 reported data sets on performed or referred ultrasounds, confirmation or rule out was possible in 83.4% (*n* = 1348) of ultrasounds. PCPs reported incidental findings in 13.3% (*n* = 215) of ultrasounds, and further imaging was needed in 7.6% (*n* = 123) of ultrasounds (for further details on general outcome and demographics see supplementary Table 1).

For referred ultrasounds, data on the general outcome was often missing: In 52.4% of referred ultrasounds, PCPs did not report whether the ultrasound confirmed or ruled out the suspected diagnosis. Data on incidental findings was also missing for 52.7% of referred ultrasounds and on need for further imaging for 59.5%. Because over half the data was missing for these categories, supplementary Table 1 does not compare ultrasounds performed or referred by PCPs. Age and gender of patients was similar, whether PCPs performed the ultrasound or made a referral.

### Main clinical indications

**Fig. 2:**
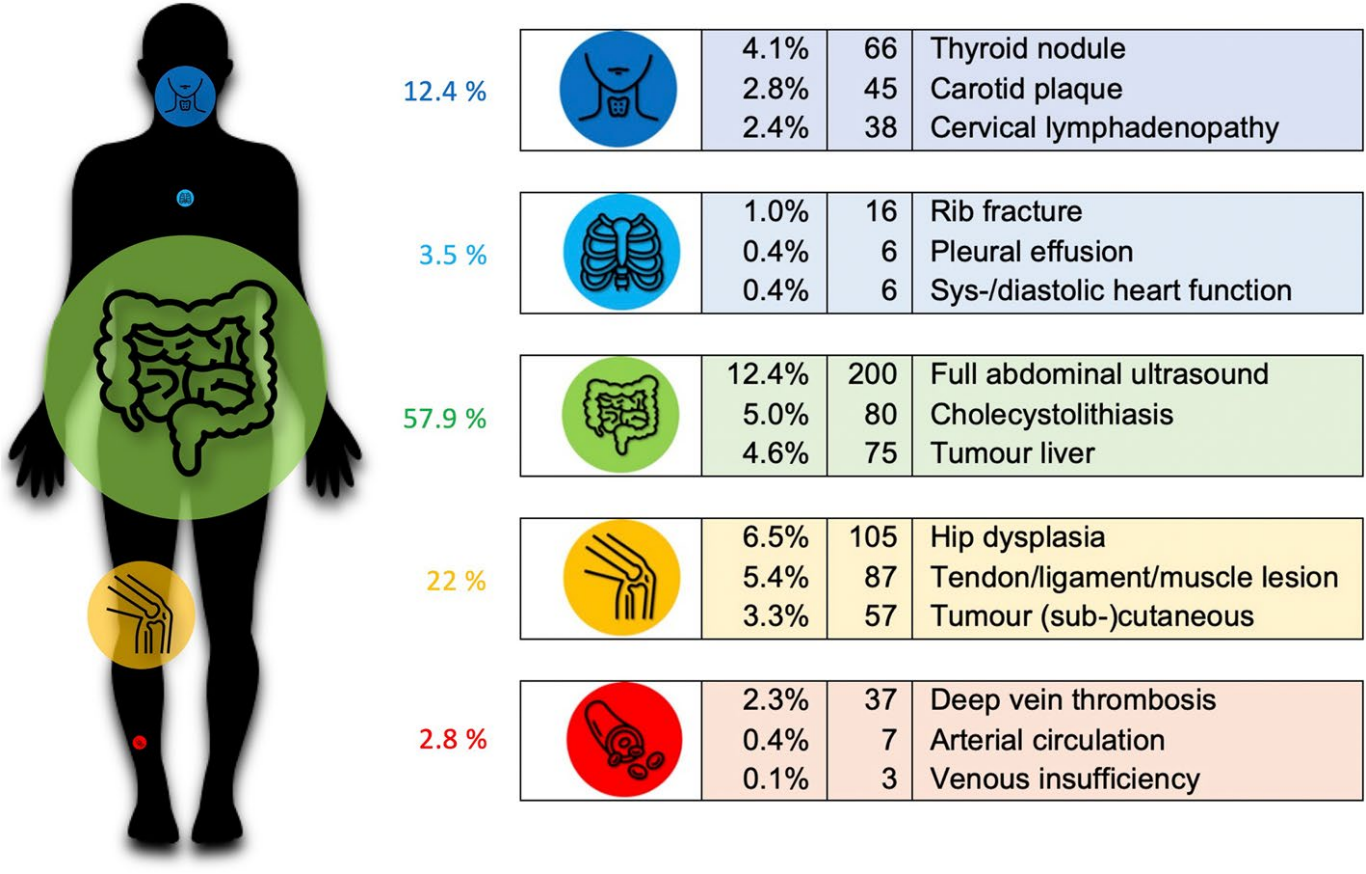
Percentages of indicated scans indicated per region and the most frequently reported indications per region. Percentage figures of the indications refer to the total number of 1616 scans

For this analysis we used all available data sets (*n* = 1616) provided by respondent group 2 and 3. Most indications were reported in the abdominal region (57.9%), followed by musculoskeletal (22%), head/neck (12.4%), thorax (3.5%), and vascular (2.8%). In 1.4% of ultrasounds, PCPs did not report the region of interest. Figure 2 shows percentages of indicated scans indicated per region and the most frequently reported indications per region.

Overall, full abdominal ultrasound (12.4%) was the most reported main indication, followed by screening for hip dysplasia in new-borns (6.5%), which was mainly (62.9%, *n* = 66) reported by paediatricians. Other common indications were mainly in the abdominal region: cholecystolithiasis (5%); liver tumour (4.6%); kidney congestion (3.7%); bladder filling condition (3.3%); nephro-/urolithiasis (3.2%); and musculoskeletal region (tendon/ligament/muscle injuries (5.4%), cutaneous/subcutaneous tumour (3.3%). More detailed information on the frequency of indications can be found under supplementary Table 2.

### Subgroup analysis: ultrasounds performed by PCP versus referred ultrasounds

For this subgroup analysis, we only included data from respondent group 3. We further excluded all data sets reported by 9 paediatricians with access to ultrasound machines, which left us with a total of 1318 data sets that were reported by 48 GPs with access to ultrasound in their practice.

Table 3 shows examinations performed by GPs and referred examinations divided into two groups. The first group contains all sPOCUS indications (indications that were included in any Swiss POCUS training program at the time of this study). The second group contains all non-sPOCUS indications (indications that were not included in any Swiss POCUS training program at the time of this study or more comprehensive scans). GPs with access to ultrasound machines (respondent group 3 minus 9 paediatricians) scanned most of the indications themselves; 88.5% (*n* = 1144) were performed by the GPs, and 11.5% (*n* = 149) were referred; 25 data sets didn’t state if the GP had performed the ultrasound or if it was referred, so we excluded these from further analysis.

**Table 3:** sPOCUS and non-sPOCUS examinations performed or referred by GPs

sPOCUS self-performed *N* = *422* *Total of 32 different indications*	Number	Percentage	Indication	Non-sPOCUS self-performed *N* = *722* *Total of 60* *different indications*	Number	Percentage	Indication
	58	*13.7%*	Cholecystolithiasis		150	*20.8%*	Full abdominal ultrasound
	48	*11.4%*	Bladder filling condition		58	*8.0%*	Liver tumour
	46	*10.9%*	Tendon/ligament/muscle injuries		56	*7.8%*	Thyroid nodule
	44	*10.4%*	Kidney congestion		44	*6.1%*	Nephro-/urolithiasis
	38	*9.0%*	Cutaneous/subcutaneous tumour		44	*6.1%*	Carotid plaque
	29	*6.9%*	Abdominal aortic aneurysm		35	*4.8%*	Screening for hip dysplasia
	25	*5.9%*	Joint effusion/joint puncture		34	*4.7%*	Evaluation of the prostate
	22	*5.2%*	Venous thrombosis		32	*4.4%*	Cervical lymphadenopathy
	21	*5.0%*	Cholecystitis		29	*4.0%*	Cirrhosis of the liver
	19	*4.5%*	Obstructive jaundice		24	*3.3%*	Bladder tumour
	15	*3.6%*	Appendicitis		22	*3.0%*	Struma/neck tumour
	14	*3.3%*	Ascites/free fluid		20	*2.8%*	Diverticulitis
	5	*1.2%*	Systolic/diastolic heart function		18	*2.5%*	Kidney tumour
	5	*1.2%*	Arterial circulation		17	*2.4%*	Splenomegaly
	4	*0.9%*	Pleural effusion		13	*1.8%*	Rib fracture
Sum	393	*93.1%*		Sum	596	*82.5%*	
sPOCUS referred *N* = *57* *Total of 16* *different indications*	Number	Percentage	Indication	Non-sPOCUS referred *N* = *92* *Total of 30* *different indications*	Number	Percentage	Indication
	13	*22.8%*	Tendon/ligament/muscle injuries		24	*26.1%*	Full abdominal ultrasound
	10	*17.5%*	Cholecystolithiasis		8	*8.7%*	Liver tumour
	8	*14.0%*	Cutaneous/subcutaneous tumour		5	*5.4%*	Inguinal-/femoral hernia
	5	*8.8%*	Venous thrombosis		4	*4.3%*	Struma/neck tumour
	4	*7.0%*	Kidney congestion		4	*4.3%*	Thyroid nodule
	3	*5.3%*	Cholecystitis			*4.3%*	Nephro-/urolithiasis
	2	*3.5%*	Bladder filling condition		4	*4.3%*	Kidney tumour
	2	*3.5%*	Abdominal aortic aneurysm		4	*4.3%*	Lymphadenopathy extremities
	2	*3.5%*	Appendicitis		3	*3.3%*	Screening for hip dysplasia
	2	*3.5%*	Thoracic tumour		3	*3.3%*	Cervical lymphadenopathy
	1	*1.8%*	Obstructive jaundice		3	*3.3%*	Cirrhosis of the liver
	1	*1.8%*	Joint effusion/joint puncture		2	*2.2%*	Stenosis of renal arteries
	1	*1.8%*	Arterial circulation		2	*2.2%*	Sialolithiasis
	1	*1.8%*	Pleural effusion		1	*1.1%*	Splenomegaly
	1	*1.8%*	Bursitis subacromialis		1	*1.1%*	Bladder tumour
Sum	56	*98.2%*		Sum	78.3%	*72%*	

Examinations performed by GPs and referred examinations divided into two groups. The first group contains all sPOCUS indications (indications that were included in any Swiss POCUS training program at the time of this study). The second group contains comprehensive ultrasound indications and all indications not included in the Swiss POCUS training program. Percentages refer to the total number of scans per group (indicated under the category name)

Of all sPOCUS indications 88.1% (*n* = 422, 32 different indications) had been performed by the GPs and 11.9% (*n* = 57, 16 different indications) had been referred. Ultrasounds for non-sPOCUS indications were performed by GPs in 88.7% of cases (*n* = 722, 60 different indications) and referred in 11.3% (*n* = 92, 30 different indications).

Examinations performed by the GPs were sPOCUS indications in 36.9% of cases (*n* = 422; 32 different indications) and non-sPOCUS indications in 63.1% (*n* = 722; 60 different indications). Examinations referred by GPs were sPOCUS indications in 38.3% of cases (*n* = 57; 16 different indications) and were non-sPOCUS indications in 61.7% (*n* = 92; 30 different indications).

The most reported sPOCUS indications were similar across groups (referred and self-performed). Some sPOCUS indications were particularly often referred: tendon/ligament/muscle injuries (10.9% performed vs. 22.8% referred) and cutaneous/subcutaneous tumour (9.0% performed vs. 14% referred). Some sPOCUS indications were particularly often self-performed: bladder filling (11.4% performed vs. 3.5% referred) and joint effusion/puncture (5.9% performed vs. 1.8% referred).

The most reported non-sPOCUS indications were also similar across the groups of GPs who referred or performed the ultrasound. Some non-sPOCUS indications were particularly often referred: abdominal scans (20.8% performed vs. 26.1% referred) and inguinal hernia (1.5% performed vs. 5.4% referred). Some non-sPOCUS indications were particularly often self-performed: carotid plaque (6.1% performed vs. 0.0% referred); prostate evaluation (4.7% performed vs. 0.0% referred); and thyroid nodules (7.8% self-performed vs. 4.3% referred).

## Discussion

### Key results

56.5% of PCPs (*n* = 87) had access to ultrasound machines; but only 52.9% of those (*n* = 46) had completed formal training. Overall, 29.8% (*n* = 46) of participating PCPs said they had completed formal ultrasound training. 77% of the reported ultrasounds were self-performed; 27% of the reported scans (35% of all self-performed scans) were performed by PCPs with incomplete or no formal training.

The main areas of interest for ultrasound in primary care in Switzerland were the abdominal (57.9%) and the musculoskeletal (22%) region. A sub-analysis showed that 63.1% of the indications for ultrasound examinations that PCPs performed were non-sPOCUS indications.

The proportion of referrals were similar for sPOCUS (11.9%) and non-sPOCUS (11.3%) indications, but GPs especially often referred some sPOCUS musculoskeletal ultrasounds (e.g. tendon/ligament/muscle injuries, cutaneous/subcutaneous tumour).

### Interpretation and comparison to existing literature

We found that 56.5% of PCPs that responded to our baseline questionnaire (respondent group 1, 2 and 3) had access to ultrasound machines. This matches the result of 49% of Swiss PCPs using ultrasound from the 2020 study from Touhami et al. [15]. Like previous studies, we found PCPs often use ultrasound as a bedside tool for immediate diagnostics [1–5]. PCPs with access to ultrasound machines perform most of the exams they indicate themselves. These exams usually provide immediate and often conclusive results: in our study, 83.4% (*n* = 1348) of reported scans either confirmed or ruled out the suspected diagnosis. Our findings add evidence to the argument that bedside ultrasounds lead to faster diagnostic workup, improves clinical decision making and can help PCPs make more rational decisions about allocating healthcare resources [7, 9–12].

However, among those PCPs (56.5%; *n* = 87), who have access to ultrasound machines, only 52.9% (*n* = 46) have completed formal training. As a result, 35% of the self-performed ultrasounds in our study were performed by PCPs with no or incomplete formal training. These findings along with the established benefit of ultrasound in primary care suggest that Swiss PCPs need more training in ultrasound and especially in POCUS.

Almost 80% of the reported scans in our study concerned the abdominal (57.9%) or the musculoskeletal (22%) region, which aligns with results of a Swiss survey analysed in 2020 (69% abdominal and 8% musculoskeletal) and with those of two Scandinavian studies also published in 2020. Our results differ only regarding obstetric and cardiac ultrasound which, in Switzerland, are almost always performed by gynaecologists and cardiologists [7, 15, 20]. We thus argue that POCUS training programs for PCPs should focus mainly on the abdominal and musculoskeletal regions.

In our sub-analysis, we further differentiated clinical questions that were performed or referred more or less often by GPs. This analysis included only data sets reported by GPs with access to ultrasound machines (respondent group 3 minus 9 paediatricians). The majority of all scans performed in this subgroup were non-sPOCUS indications. This finding was contradictory to our expectations since the POCUS approach is inherently very close to typical applications for ultrasound in primary care and the percentage of POCUS examinations seems to be higher in other European countries [7]. We assume the only recent introduction of the POCUS concept in Switzerland and the lack of a specific POCUS training-program for Swiss PCPs to be the reason for this finding [13, 22]. Furthermore, studies from other countries suggest a higher diagnostic accuracy for POCUS scans than more comprehensive scans when performed by PCPs [4]. Further research is needed to assess whether this also applies to Switzerland where comprehensive ultrasounds are often performed by PCPs.

In the same sub-analysis, we found that the percentage of sPOCUS scans was almost equal and relatively low for both performed and referred scans, most likely due to the same reasons as stated above. Our results showed that some sPOCUS indications in the musculoskeletal region were particularly often referred. We thus propose the inclusion of those ultrasound indications in future POCUS training programs aimed at PCPs.

## Limitations and strengths

Our study has four major limitations.

First, we collected data solely within the Sentinella-Network from Swiss PCPs, so our results may not be generalizable outside of Switzerland. For example, in Switzerland, unlike many other countries, obstetric and cardiac ultrasounds are almost exclusively performed by gynaecologists and cardiologists.

Second, because PCPs in the Sentinella network usually submit data daily or weekly, we received data about many of the referred examinations before PCPs received their results. We thus could not compare the quality of results of performed and referred ultrasound scans in this study (see supplementary Table 1). Further research with a longer response latency between ultrasound referral and data collection is needed to determine the value of self-performed ultrasound scans in comparison to referred scans.

Third, interestingly PCPs with access to ultrasound machines (respondent group 3) submitted far more data sets on ultrasounds than PCPs without access (respondent group 2) (1425 = 25 data sets per PCP vs. 191 = 6.2 data sets per PCP). This could be due to an underreporting of PCPs without access to ultrasound machines with less interest in the topic of this study. Another explanation could be, that PCPs with access to ultrasound machines have an higher affinity to the study’s topic and are aware of more possible use ceases, which likely leads to more indicated ultrasound examinations and probably to a higher reporting rate. A drop out analysis showed that PCPs that collected data (respondent group 2 and 3) had access to ultrasound in 64.8%, while PCPs that didn’t collect data (respondent group 1) had access to ultrasound in 45.5%. All of the above indicates a possible selection bias, since PCPs with access to ultrasound machines and the data sets reported by them are overrepresented in this study. This bias is only relevant for the outcome of all indications of ultrasound scans performed or referred over a period of one year, since respondent group 1 and 2 were not included for the subgroup analysis. Also, a comparison between submitted data between respondent group 2 and 3 showed that they indicated similar indications with similar proportional distribution.

Fourth, PCPs with access to ultrasound machines (respondent group 3) also performed most exams themselves (77% (*n* = 1245) of all data sets). We had far fewer data sets on referred examinations (21.2%; *n* = 343). In our sub-analysis of GPs with access to ultrasound machines (respondent group 3 minus 9 paediatricians), only 11.5% of data sets were on referred examinations. This and the lack of follow-up data on referred examinations (as stated above) precluded in-depth comparison of performed and referred examinations. Studies aiming at comparing outcomes of referred ultrasounds vs. self-performed ultrasounds should take into consideration the difficulty of collecting data and follow-up data on referred ultrasounds.

A major strength of this study were high response rates (81.9% for the baseline questionnaire and 46.8% for the main data collection). We were able to collect a relatively big data set compared to previous studies [7, 18]. Also, the above-mentioned, short response latency was also a strength of this study since we assume immediate data collection to provide more precise and detailed answers than retrospective data collection over a period of e.g. several months.

## Conclusions

Most Swiss PCPs had access to ultrasound equipment and performed a majority of both sPOCUS and non-sPOCUS scans themselves, often without or with incomplete training. This reflects the fact that POCUS was only recently introduced in Switzerland. There is a need for easily accessible POCUS training programs aimed at PCPs in Switzerland.

Training courses for PCPs should focus on abdominal and musculoskeletal ultrasound, because these were the most common sites for scans, and because some sPOCUS musculoskeletal examinations showed a particularly high percentage of referral.

### Supplementary Information

Supplementary Material 1.

Supplementary Material 2.

Supplementary Material 3: Supplementary Table 1. Demographics and general outcomes of all 1616 ultrasounds. Legend: -

Supplementary Material 4: Supplementary Table 2. The 20 most frequently reported indications. Legend: Percentages refer to the total number of 1616 scans.

## Data Availability

The datasets used and/or analysed during the current study are available from the corresponding author on reasonable request.

## Abbreviations

PCPs: Primary care physicians
POCUS: Point-of-care ultrasound
sPOCUS: SwissPOCUS
non-sPOCUS: Non-swissPOCUS
GPs: General practitioners
