# Supplementary material for: The use of diagnostic ultrasound by primary care physicians in Switzerland – a cross-sectional study
Source: BMC Prim Care. 2024 Jul 6;25:246. doi: 10.1186/s12875-024-02491-5 (PMC11227144; doi:10.1186/s12875-024-02491-5)
Supplement: Supplementary file 1 — Supplementary Material 1. [file 12875_2024_2491_MOESM1_ESM.docx]

*The following questionnaire is an English translation of the original questionnaire, which was presented to the participating physicians in German or French trough www.* *surveymonkey.com.*

1. Please enter your Sentinella doctor code. (Used to assign the data collected): ________
2. Please check the appropriate box

| There is always an ultrasound machine ready for use in my usual treatment room. |  |
| --- | --- |
| I have access to an ultrasound machine in my practice, but I either have to bring it into the room or take the patient to another room. |  |
| I have access to an ultrasound machine if required, but only outside my practice. |  |
| I don't have access to an ultrasound machine. |  |

1. Please indicate your employment percentage: ________
2. How many sonographies do you carry out per week?

| 0-5 |  |
| --- | --- |
| 6-10 |  |
| 11-15 |  |
| >15 |  |

1. Have you regularly scheduled a fixed amount of time for elective sonographies?

| Yes |  |
| --- | --- |
| No |  |

1. If question 5 was answered with yes: How many hours per week have you scheduled for elective sonographies? ________
2. Please check the appropriate box

| I treat exclusively pediatric patients (<16 yrs.)  *🡪 continue with QUESTION 10* |  |
| --- | --- |
| I only treat adult patients (>16 yrs.)  *🡪 continue with QUESTION 8 and skip QUESTION 10 and 11* |  |
| I treat both pediatric and adult patients  *🡪 continue with QUESTION 8* |  |

1. Please indicate your level of training in adult sonography (multiple answers possible)

| Certificate of competence in abdominal sonography (completed final course and regular recertification) |  |
| --- | --- |
| Completed advanced course in abdominal sonography |  |
| Completed basic course in abdominal sonography |  |
| No formal training. Lots of practical experience. |  |
| No formal training. Little practical experience. |  |
| No formal training. No practical experience. |  |
| Other: |  |

1. In the case of an adult patient (>16 yrs.): For which of the following pathologies do you have the confidence to make a sonographic diagnosis (or at least rule out) yourself in your practice? Which of the pathologies are you unable to assess and refer the patient for further imaging?

Please check the appropriate box:

|  | I am able to diagnose | I am able to rule out | I am not able to asses |
| --- | --- | --- | --- |
| Free abdominal fluid/ascites |  |  |  |
| Cholecystolithiasis |  |  |  |
| Cholecystitis |  |  |  |
| Cholestasis |  |  |  |
| Pancreatitis |  |  |  |
| Liver cirrhosis |  |  |  |
| Liver tumour |  |  |  |
| Hepato-/splenomegaly |  |  |  |
| Renal congestion |  |  |  |
| Urolithiasis |  |  |  |
| Residual urine |  |  |  |
| Prostatic hyperplasia |  |  |  |
| Abdominal tumour |  |  |  |
| Ovarian cyst |  |  |  |
| Appendicitis |  |  |  |
| Diverticulitis |  |  |  |
| Pneumonia |  |  |  |
| Pleural effusion |  |  |  |
| Pericardial effusion |  |  |  |
| Abdominal aortic aneurysm |  |  |  |
| Signs of heart failure (inferior vena cava/liver congestion) |  |  |  |
| deep vein thrombosis |  |  |  |
| Soft tissue pathology (e.g. lipoma, cyst, abscess, malignancy) |  |  |  |
| Thyroid nodules |  |  |  |
| Joint effusion |  |  |  |
| Fractures |  |  |  |
| Foreign body |  |  |  |
| Other: |  |  |  |

1. Please indicate your level of training in sonography for paediatric patients (multiple answers possible)

| POCUS for paediatrics |  |
| --- | --- |
| Hip sonography in new-borns and infants |  |
| No formal training. Lots of practical experience. |  |
| No formal training. Little practical experience. |  |
| No formal training. No practical experience. |  |
| Other: |  |

1. In the case of a paediatric patient (<16 years): For which of the following pathologies do you feel confident in making a sonographic diagnosis yourself in the practice? For which would you at least exclude? Which of the pathologies are you unable to assess and refer the patient for further imaging?

Please check the appropriate box:

|  | I am able to diagnose | I am able to rule out | I am not able to asses |
| --- | --- | --- | --- |
| Hepato-/splenomegaly |  |  |  |
| Free fluid in body cavities |  |  |  |
| Invagination, malrotation |  |  |  |
| Appendicitis and other acute inflammatory bowel infections |  |  |  |
| Lung consolidations |  |  |  |
| Urinary outflow obstruction (hydronephrosis, megaureter, urinary retention, residual urine) |  |  |  |
| Fractures of long bones and skull calvaria |  |  |  |
| Joint effusion (hip, knee, elbow) |  |  |  |
| Abscess in skin and soft tissue |  |  |  |
| Lymphadenopathy |  |  |  |
| Space-occupying lesions (DD solid or cystic) |  |  |  |
| Foreign body |  |  |  |
| Hip dysplasia (hip sonography according to Graf) |  |  |  |
| Others: |  |  |  |

1. For which of the following indications/questions do you consider certification by means of a POCUS certificate of competence in family medicine to be useful? (multiple answers possible)

Please check the appropriate box:

| Free abdominal fluid/ascites |  |
| --- | --- |
| Cholecystolithiasis |  |
| Cholecystitis |  |
| Cholestasis |  |
| Pancreatitis |  |
| Liver cirrhosis |  |
| Liver tumour |  |
| Hepato-/splenomegaly |  |
| Renal congestion |  |
| Urolithiasis |  |
| Residual urine |  |
| Prostatic hyperplasia |  |
| Abdominal tumour |  |
| Ovarian cyst |  |
| Appendicitis |  |
| Diverticulitis |  |
| Pneumonia |  |
| Pleural effusion |  |
| Pericardial effusion |  |
| Abdominal aortic aneurysm |  |
| Signs of heart failure (inferior vena cava/liver congestion) |  |
| deep vein thrombosis |  |
| Soft tissue pathology (e.g. lipoma, cyst, abscess, malignancy) |  |
| Thyroid nodules |  |
| Joint effusion |  |
| Fractures |  |
| Foreign body |  |
| Other: |  |

1. The expected requirements for obtaining a POCUS in Family Medicine are listed below:

- 1x basic abdomen course (21h)

- 100 supervised and 100 non-supervised sonographies

- 1x POCUS primary care course (approx. 8-16 hours)

- No final examination

- Recertification every 5 years

Let's assume you are at the beginning of your career as a GP and have not completed a "Certificate of Competence in Abdominal Sonography". Would you consider acquiring a POCUS certificate in family medicine?

Please check the appropriate box:

| Yes |  |
| --- | --- |
| Rather yes |  |
| Maybe |  |
| Rather no |  |
| No |  |
