# Supplementary material for: The use of diagnostic ultrasound by primary care physicians in Switzerland – a cross-sectional study
Source: BMC Prim Care. 2024 Jul 6;25:246. doi: 10.1186/s12875-024-02491-5 (PMC11227144; doi:10.1186/s12875-024-02491-5)
Supplement: Supplementary file 2 — Supplementary Material 2. [file 12875_2024_2491_MOESM2_ESM.docx]

The following questionnaire is an English translation of the original questionnaire, which was presented to the participating physicians in German or French through the Sentinella data collection tool.

1. Patient’s year of birth
2. Patient’s gender
3. Ultrasound performed by yourself: Yes/No
4. Urgency of the ultrasound (regardless of referred or self-performed)
   1. Emergency (must be carried out on the same day)
   2. Urgent (to be performed promptly within 1-3 days)
   3. Non-urgent (to be performed at a later date (>3 days))
5. Please select the suspected diagnosis/main question and up to two secondary questions: (The list of questions is sorted by body region. Please leave the fields you do not need empty)
   1. Head/neck
      1. Sialadenitis
      2. Sialolithiasis
      3. Lymphadenopathy
      4. Thyroid nodule
      5. Goitre/tumour neck
      6. Carotid stenosis
      7. Carotid plaque
      8. Volume status (jugular vein congestion)
      9. Other
   2. Shoulders/upper extremity
      1. Fracture
      2. Rotator cuff lesion
      3. Joint effusion/joint puncture
      4. DVT
      5. Arterial circulation
      6. Venous/arterial puncture
      7. Tendon/ligament/muscle injuries
      8. Other
   3. Thorax
      1. Pleural effusion
      2. Pneumothorax
      3. Pneumonia
      4. Pericardial effusion
      5. Systolic/diastolic cardiac function
      6. Cardiac wall motion abnormalities
      7. Heart valve insufficiency/stenosis
      8. Volume status (inferior vena cava)
      9. Rib fracture
      10. Other
   4. Abdomen
      1. Free fluid/ascites
      2. Free air
      3. Liver tumour
      4. Liver cirrhosis
      5. Cholecystitis
      6. Cholecystolithiasis
      7. Obstructive jaundice
      8. Pancreatitis
      9. Pancreatic tumour
      10. Abdominal aortic aneurysm
      11. Splenomegaly
      12. Kidney congestion
      13. Nephro/urolithiasis
      14. Renal tumour
      15. Bladder tumour
      16. Bladder filling condition
      17. Ultrasound screening in pregnancy
      18. EUG
      19. Ovarian tumour
      20. Uterine tumour
      21. Prostate evaluation
      22. Testicular torsion
      23. Orchitis/epididymitis
      24. Appendicitis
      25. Diverticulitis
      26. Ileus
      27. Invagination, malrotation
      28. Intestinal tumour
      29. Psoas haematoma
      30. Full abdominal ultrasound
      31. Other
   5. Groin/lower extremity
      1. Lymphadenopathy
      2. Inguinal/femoral hernia
      3. Venous thrombosis
      4. Arterial circulation
      5. Baker's cyst
      6. Compartment syndrome
      7. Fracture
      8. Tendon/ligament/muscle injuries
      9. Joint effusion/joint puncture
      10. Screening for hip dysplasia in new-born
      11. Other
   6. Integument
      1. Cutaneous/subcutaneous tumour
      2. Foreign body
      3. Other
6. Other suspected diagnosis/main question?
7. If ultrasound was performed by yourself: Duration of the ultrasound in minutes?
8. if you performed the ultrasound yourself: How was the ultrasound billed?
   1. As ultrasound examination according to Tarmed
   2. As a clinical examination according to Tarmed
   3. The examination was not billed

Questions 9-11 refer to the findings and the consequences of the sonography. If the result is not yet available at the time of recording, please leave questions 9-11 unanswered

1. Result of the ultrasound:
   1. Main question/suspected diagnosis confirmed
   2. Main question/suspected diagnosis ruled out
   3. Main question cannot be conclusively assessed
2. Were there any incidental findings during the ultrasound examination?
   1. No
   2. Yes (and which?)
3. further imaging necessary following ultrasound examination?
   1. No
   2. Referral for repeat ultrasound
   3. Referral for conventional X-ray/CT/MRI/scintigraphy (etc.)
   4. Other: _______
