# Supplementary material for: The use of diagnostic ultrasound by primary care physicians in Switzerland – a cross-sectional study
Source: BMC Prim Care. 2024 Jul 6;25:246. doi: 10.1186/s12875-024-02491-5 (PMC11227144; doi:10.1186/s12875-024-02491-5)
Supplement: Supplementary file 3 — Supplementary Material 3: Supplementary Table 1. Demographics and general outcomes of all 1616 ultrasounds. Legend: - [file 12875_2024_2491_MOESM3_ESM.docx]

**Supplementary Table 1**

**Title:** Demographics and general outcomes of all 1616 ultrasounds

| Number of ultrasounds |  | 1616 | *100 %* |
| --- | --- | --- | --- |
| Average age of patients *(years)* |  | 41 | *-* |
| Gender of patients | Female | 784 | *48.5 %* |
|  | Male | 832 | *51. 5 %* |
| Suspected diagnosis | Confirmed | 638 | *39.5 %* |
|  | Ruled out | 710 | *43.9 %* |
|  | Not conclusive | 75 | *4.6 %* |
|  | Unknown | 193 | *11.9 %* |
| Incidental finding | Yes | 215 | *13.3 %* |
|  | No | 1185 | *73.3 %* |
|  | Unknown | 216 | *13.4 %* |
| Further imaging | Yes | 123 | *7.6 %* |
|  | No | 1207 | *74.7 %* |
|  | Unknown | 286 | *17.7 %* |

**Legend:** -
