# Supplementary material for: The use of diagnostic ultrasound by primary care physicians in Switzerland – a cross-sectional study
Source: BMC Prim Care. 2024 Jul 6;25:246. doi: 10.1186/s12875-024-02491-5 (PMC11227144; doi:10.1186/s12875-024-02491-5)
Supplement: Supplementary file 4 — Supplementary Material 4: Supplementary Table 2. The 20 most frequently reported indications. Legend: Percentages refer to the total number of 1616 scans. [file 12875_2024_2491_MOESM4_ESM.docx]

**Supplementary Table 2**

**Title:** The 20 most frequently reported indications

| Rank | Number | Percentage | Region | Indication |
| --- | --- | --- | --- | --- |
| *1* | 200 | *12.4 %* | Abdomen | Full abdominal ultrasound |
| *2* | 105 | *6.5 %* | Musculoskeletal | Screening for hip dysplasia in new-born |
| *3* | 87 | *5.4 %* | Musculoskeletal | Tendon/ligament/muscle injuries |
| *4* | 80 | *5.0 %* | Abdomen | Cholecystolithiasis |
| *5* | 75 | *4.6 %* | Abdomen | Liver tumour |
| *6* | 66 | *4.1 %* | Head/neck | Thyroid nodule |
| *7* | 59 | *3.7 %* | Abdomen | Kidney congestion |
| *8* | 54 | *3.3 %* | Abdomen | Bladder filling condition |
| *9* | 54 | *3.3 %* | Musculoskeletal | Cutaneous/subcutaneous tumour |
| *10* | 52 | *3.2 %* | Abdomen | Nephro-/urolithiasis |
| *11* | 45 | *2.8 %* | Head/neck | Carotid plaque |
| *12* | 38 | *2.4 %* | Head/neck | Cervical lymphadenopathy |
| *13* | 37 | *2.3 %* | Vascular | Venous thrombosis |
| *14* | 37 | *2.3 %* | Musculoskeletal | Joint effusion/joint puncture |
| *15* | 36 | *2.2 %* | Abdomen | Evaluation of the prostate |
| *16* | 34 | *2.1 %* | Abdomen | Cirrhosis of the liver |
| *17* | 33 | *2.0 %* | Head/neck | Struma/neck tumour |
| *18* | 32 | *2.0 %* | Abdomen | Abdominal aortic aneurysm |
| *19* | 27 | *1.7 %* | Abdomen | Obstructive jaundice |
| *20* | 27 | *1.7 %* | Abdomen | Inguinal/femoral hernia |

**Legend:** Percentages refer to the total number of 1616 scans.
